# Systematic evaluation of the teaching effectiveness of flipped classroom mode in undergraduate preventive medicine courses in China

**DOI:** 10.3389/fpubh.2026.1896815

**Published:** 2026-08-03

**Authors:** Hui Zheng, Yun Ouyang, Yifei Zeng, Xinyu He, Liping Song, Xiangyuan Yu

**Affiliations:** 1 Department of Epidemiology and Health Statistics, School of Public Health, Guilin Medical University, Guilin, China; 2 Guangxi Key Laboratory of Diabetic Systems Medicine, Guilin Medical University, Guilin, China; 3 School of Humanities and Management, Guilin Medical University, Guilin, China

**Keywords:** effectiveness, flipped classroom, meta-analysis, preventive medicine courses, traditional classroom

## Abstract

**Objective:**

To systematically evaluate the application effect of flipped classroom (FC) teaching mode in preventive medicine courses in Chinese medical colleges and universities.

**Methods:**

A systematic search was conducted in PubMed, Web of Science, China National Knowledge Infrastructure (CNKI), etc. to search for reports on the FC teaching model in preventive medicine courses. A quantitative system evaluation was conducted to assess the teaching effectiveness of FC with multiple indicators such as exam score, learning interest, and self-learning and thinking abilities.

**Results:**

A total of 28 articles were included, including 2,135 students in the flipped classroom group and 2,259 students in the LBL group. It showed that compared with the lecture-based learning (LBL) group, the FC can significantly improve students’ exam scores for both theoretical and practical components, their quantitatively synthesized SMDs were 0.99 (95% CI: 0.65–1.33) and 0.50 (95% CI: 0.30–0.70), respectively. Meanwhile, learning interest (RR = 1.42, 95%CI:1.33–1.53), independent learning and thinking ability (RR = 1.51, 95%CI: 1.31–1.73), cooperation and communication skills (RR = 1.60, 95%CI: 1.28–2.01), problem analysis and solving ability (RR = 1.47, 95%CI: 1.13–1.91), and teacher-student interaction (RR = 1.35, 95%CI: 1.10–1.66), the differences are statistically significant (*p* < 0.05).

**Conclusion:**

The FC teaching model has shown significant advantages in the teaching of preventive medicine courses in Chinese higher medical colleges. In view of this, schools can reasonably apply it in relevant course teaching according to resources such as faculty and facilities.

## 1 Introduction

Preventive medicine, as a core component of the medical education system, encompasses courses—such as epidemiology, health statistics, and environmental hygiene—that are highly theoretical and practical (1). In the traditional lecture-based learning (LBL) mode, students are often passive recipients of information, with limited teacher–student interaction, making it challenging for students to integrate abstract public health theories with complex disease prevention and control practices, which leads to a lack of initiative in learning, restricted critical thinking, and a weakened ability to solve real-world public health problems (2). As reforms in modern medical education continue to advance, it is imperative to explore innovative teaching models aligned with the characteristics of preventive medicine.

Lage MJ et al., who were at the University of Miami in the United States, first proposed the concept of the “flipped classroom “(FC), which has since been widely applied across multiple disciplines (3). FC is regarded as a blended learning model that inverts the traditional teaching process (4). The core idea of FC is to shift the delivery of knowledge to before class, allowing students to independently acquire it through activities such as watching instructional videos and reading the literature, while classroom time is dedicated to deeper learning activities, including teacher–student interaction, case discussions, and problem-solving (5). This student-centered model promotes personalized learning and collaborative inquiry, and is believed to help develop students’ self-directed learning ability, clinical reasoning skills, and teamwork spirit (6). It is thus expected to compensate for the shortcomings of LBL teaching. Currently, the FC model has been widely adopted and promoted in various areas of medical education in China, including preventive medicine.

In China, a number of studies have explored the effectiveness of implementing FC in preventive medicine courses by comparing students’ theoretical exam scores, professional skill scores, and questionnaire surveys on learning interest and satisfaction (7–34). However, the conclusions drawn from these studies are not entirely consistent, and due to factors such as small sample sizes and course heterogeneity, it is difficult for the results of any single study to comprehensively and objectively reflect the true effectiveness of this model in preventive medicine teaching. To date, there has been a lack of large-scale quantitative combined analysis on the effectiveness of FC teaching for preventive medicine courses in China.

Therefore, this study conducted a systematic evaluation to address two research questions: (1) whether FC can significantly improve the exam scores of undergraduate students in preventive medicine, covering both theoretical and practical aspects; and (2) what impact FC has on subjective indicators such as students’ learning interest, independent learning and thinking abilities, cooperation and communication skills. The findings thus provide evidence-based support for the teaching reform of courses related to preventive medicine.

## 2 Materials and methods

### 2.1 Literature inclusion and exclusion criteria

The inclusion criteria were as follows: (1) The study participants were students enrolled in preventive medicine courses at Chinese medical colleges or universities; (2) The trial consisted of implementing the FC teaching model in preventive medicine courses, such as epidemiology, health statistics, and occupational health and medicine; (3) The control group received traditional LBL instruction; (4) At least one of the following outcome indicators was reported: theoretical exam scores, learning interest, independent learning and thinking ability, cooperative communication ability, analytical and problem-solving ability, teacher-student interaction, etc.; (5) The study was designed as a RCT or a quasi-experimental study with a concurrent control group.

The exclusion criteria were as follows: (1) Reports not published in Chinese or English; (2) Reviews, abstracts, dissertations, and news reports, systematic reviews, meta-analyses; (3) The trial measures do not reflect the core characteristics of FC method or lack of single arm research on traditional teaching control group; (4) Incomplete data or the absence of key outcome indicators; (5) Duplicate publications.

### 2.2 Literature screening and data extraction

Literature was screened strictly according to the pre-established inclusion and exclusion criteria using the PICOS framework. A systematic search of PubMed, Web of Science, China National Knowledge Infrastructure (CNKI) and Wanfang Data Knowledge Service Platform was conducted to collect RCTs and quasi-experimental studies on the application of FC in the teaching of preventive medicine courses in China. The search period spanned from the inception of each database to April 2026. Search terms included: “flipped classroom,” “preventive medicine,” “epidemiology,” “health statistics,” “environmental health,” “occupational health and occupational medicine,” “teaching,” “education,” etc. The complete search strategies for each database, are provided in Supplementary material.

After removing duplicates using EndNote, two researchers independently screened the titles and abstracts for initial screening, excluding obviously irrelevant studies; subsequently, the full texts of the initially screened studies were retrieved and further assessed, mainly to exclude studies that were not RCTs or quasi-experimental studies, did not involve preventive medicine courses, lacked a control group, or failed to provide extractable data on outcome indicators (such as theoretical grades, practical skills, self-directed learning ability scores, etc.). Reference lists were also screened to identify additional eligible studies. Finally, the screening results were cross-checked, and any discrepancies were resolved through discussion or adjudication by a third researcher to finalize the set of studies meeting the inclusion criteria, and the Cochrane bias risk assessment tool was used to evaluate its methodological quality.

Data were extracted using a pre-designed Excel spreadsheet, including first author, publication year, course name, study participants, sample size (trial group/control group), teaching mode (trial group/control group), outcome indicators and their reported data (sample size, mean, standard deviation), etc.

### 2.3 Methodological quality assessment

The methodological quality of the included RCTs was assessed using RevMan 5.4 software, based on the “Risk of Bias Assessment Tool” (RoB) recommended by the Cochrane Handbook. This tool covers the following domains: random sequence generation, allocation concealment, subject and personnel blinding, outcome evaluation blinding, incomplete outcome data, selective reporting, and other bias items. Two researchers independently performed the quality assessment and cross-checked the results.

### 2.4 Statistical analysis

The meta-analysis was conducted using Stata 12.0 software. The standardized mean difference (SMD) was used as the effect measure for continuous variables, and the relative risk (RR) was used for binary variables, with the corresponding 95% confidence interval (CI) calculated. For the ordinal assessment of outcomes such as learning interest and self-directed learning ability (such as “efficient, effective, ineffective”), the “efficient” and “effective” categories were combined and converted into a binary outcome. Heterogeneity among studies was assessed using Cochran’s Q test. If *p* > 0.1 and *I^2^* < 50%, low heterogeneity was indicated, and a fixed effects model is used for meta-analysis; If *p* ≤ 0.1 or *I^2^* ≥ 50%, significant heterogeneity was indicated, and a random effects model is used for meta-analysis. Begg’s funnel plot is used to assess publication bias, and the leave-one-out method was used for sensitivity analysis to evaluate the robustness of the research findings. A two sides *p* < 0.05 was considered statistically significant.

## 3 Results

### 3.1 Meta-analysis

#### 3.1.1 Literature search

**Figure 1:**
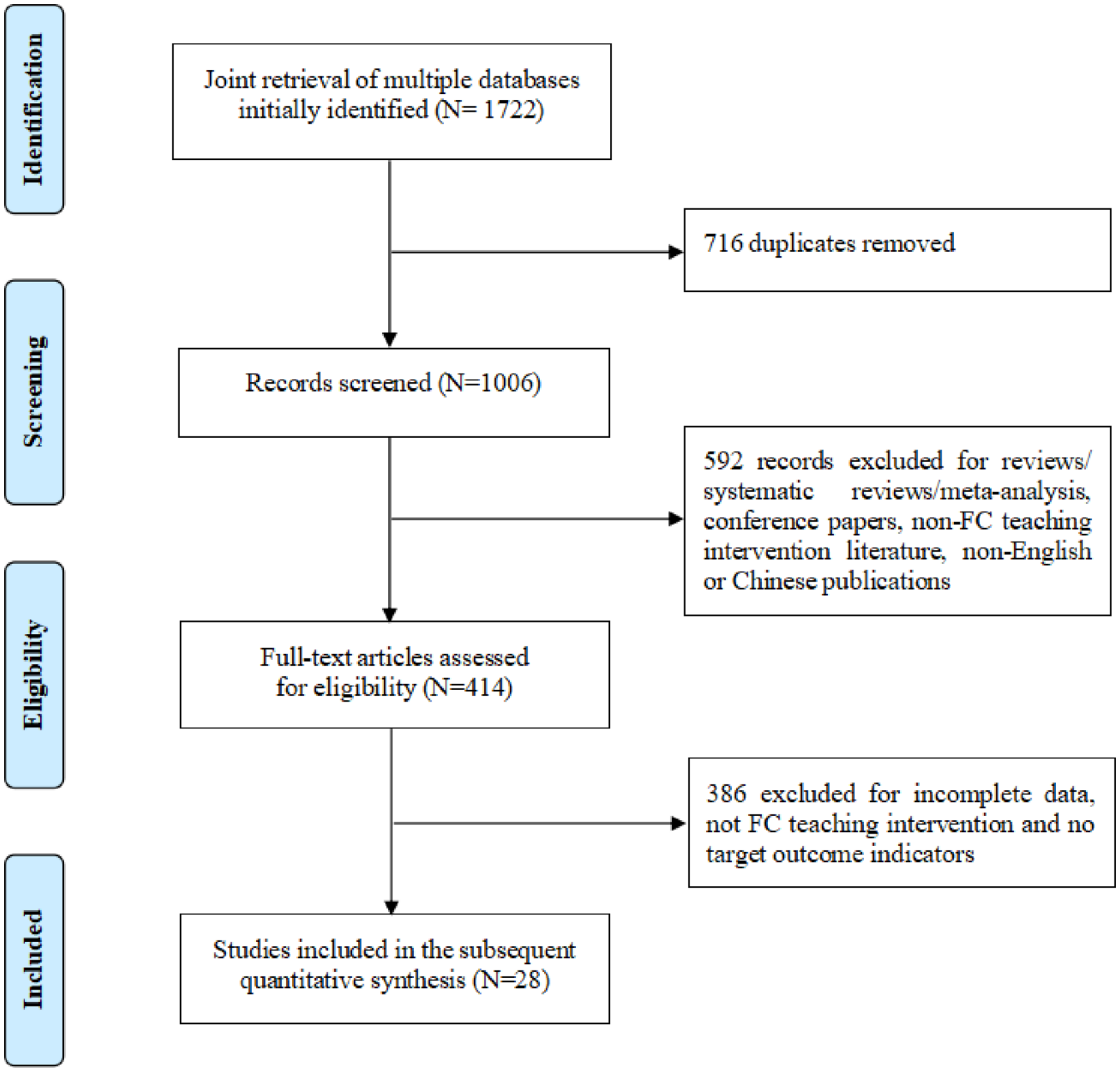
Flowchart of screening process for eligible studies.

Based on the literature screening described above, this study initially retrieved 1,722 relevant records. After removing duplicates using EndNote, 1,006 records remained; subsequently, by screening titles and abstracts, a total of 592 records were excluded, including, reviews, systematic reviews, meta-analysis, and conference papers. Further full-text review led to the exclusion of 386 articles due to reasons such as incomplete data, absence of FC teaching trial, or lack of target outcome indicators. Finally, 28 eligible studies (12 RCTs and 16 quasi-experimental studies) were included (Figure 1), with a cumulative sample size of 4,394, comprising 2,135 in the FC group and 2,259 in the traditional LBL teaching group. The basic characteristics of included studies are shown in Table 1.

**Table 1:** Basic characteristics of included studies.

Study	Year	Number		Student major	Trial group	Control group	Category	Study type		Outcomes
		FC	LBL	FC	LBL					
Liu HY et al (7)	2025	57	54	Medical Information Engineering	Medical Imaging	FC	LBL	Theoretical+Practical Course	Quasi-trial	①②,③,④,⑤
Jiang Y et al (8)	2025	243	197	Clinical Medicine	Clinical Medicine	O-PIPAS-FC	LBL + CBL	Practical Course	Quasi-trial	①
Ai ZQ et al (9)	2024	179	174	Preventive Medicine	Preventive Medicine	FC	LBL	Practical Course	Quasi-trial	①
			175						Non-randomized historical cohort comparison by academic year (2019 versus 2017/2018)	
Duan PF et al (10)	2023	141	148	Clinical Medicine	Clinical Medicine	FC + CBL	LBL	Theoretical course	RCT	①
Li CL et al (11)	2023	117	130	Clinical Medicine	Clinical Medicine	FC + CBL	LBL	Theoretical course	Quasi-trial	①,②,③,④,⑤
Li JS et al (12)	2023	74	88	Nursing	Nursing	FC + CBL	LBL	Theoretical course	Quasi-trial	①,②,③,⑤
Jiang CL et al (13)	2023	96	94	Nursing	Nursing	FC	LBL	Theoretical course	RCT	①
Zhang Y et al (14)	2023	97	95	Preventive Medicine	Preventive Medicine	FC	LBL	Theoretical course	Quasi-trial	①
Li J et al (15)	2022	87	98	Preventive Medicine	Preventive Medicine	FC	LBL	Theoretical course	Quasi-trial	①,②,③,④,⑤,⑥
Shi TL et al (16)	2022	21	19	Clinical Medicine	Clinical Medicine	FC + Rain Classroom	LBL	Theoretical course	Quasi-trial	①
Tang X et al (17)	2022	55	62	Preventive Medicine	Preventive Medicine	FC + Internet plus	LBL	Theoretical course	Quasi-trial	①
Hou HF et al (18)	2022	52	50	Preventive Medicine	Preventive Medicine	FC + Mooc	LBL	Theoretical course	RCT	①
Mu M et al (19)	2021	66	67	Preventive Medicine	Preventive Medicine	FC	LBL	Theoretical course	Quasi-trial	①
Sang JY et al (20)	2020	45	45	Preventive Medicine	Preventive Medicine	FC	LBL	Theoretical+Practical Course	Quasi-trial	①
Xiu LC et al (21)	2020	51	50	Medical Information and Information Resource Management	Medical Information and Information Resource Management	FC	LBL	Practical Course	Quasi-trial	①
Yang SG et al (22)	2020	71	71	Nursing	Nursing	FC + PBL	LBL	Theoretical course	RCT	①,②
Yu J et al (23)	2020	25	25	Preventive Medicine	Preventive Medicine	FC + Micro course	LBL	Theoretical course	RCT	①,④,⑤
Yang L et al (24)	2019	15	14	Preventive Medicine	Preventive Medicine	FC	LBL	Theoretical course	RCT	①
Jiang S et al (25)	2019	52	52	Clinical Medicine	Clinical Medicine	FC + PBL	LBL	Theoretical course	Quasi-trial	①,②,③,④,⑥
Liu X et al (26)	2019	71	39	Clinical Medicine	Clinical Medicine	FC	LBL	Theoretical course	Quasi-trial	①
Ma XH et al (27)	2019	70	69	Preventive Medicine	Preventive Medicine	FC	LBL	Practical Course	RCT	①,③,⑤
Tang SW et al (28)	2018	42	42	Preventive Medicine	Preventive Medicine	FC	LBL	Theoretical course	RCT	①,②,③,④,⑤
Guo LQ et al (29)	2018	30	30	Preventive Medicine	Preventive Medicine	FC + PBL	LBL	Theoretical course	RCT	①,②,③,④
Jia JY et al (30)	2018	60	60	Preventive Medicine	Preventive Medicine	FC	LBL	Theoretical course	RCT	①
Zhang Y et al (31)	2017	158	149	Nursing	Nursing	FC + PBL	LBL	Theoretical course	Quasi-trial	①,②,③,⑤
Mi W et al (32)	2017	56	58	Clinical Medicine	Clinical Medicine	FC + PBL	LBL	Theoretical course	Quasi-trial	①,④,⑥
Yin SY et al (33)	2017	40	40	Nursing	Nursing	FC	LBL	Theoretical course	RCT	①,②
Yang SG et al (34)	2016	64	64	Nursing	Nursing	FC	LBL	Theoretical course	RCT	①

① Examination score; ② Learning interest; ③ Independent learning and thinking ability; ④ Cooperation and communication skill; ⑤ Ability to analyze and solve problems; ⑥ Teacher-student interactions.

#### 3.1.2 Methodological quality assessment of included literature

**Figure 2:**
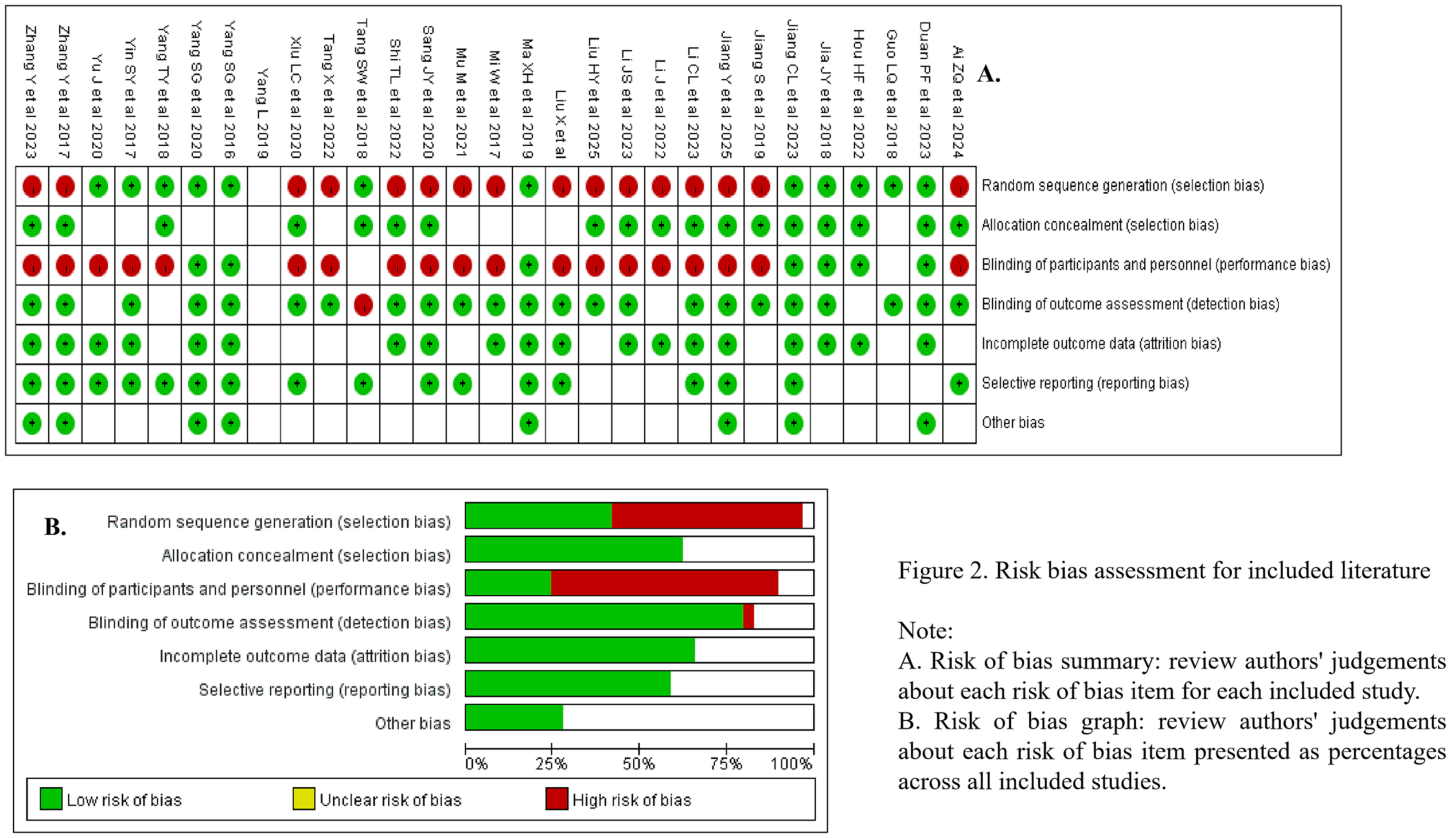
Risk bias assessment for included literature. **(A)** Risk of bias summary: Review authors’ judgments about each risk of bias item for each included study. **(B)** Risk of bias graph: Review authors’ judgments about each risk of bias item presented as percentages across all included studies.

According to the Cochrane Handbook’s recommended “Risk of Bias Assessment Tool” (RoB). The assessment addressed selection bias, performance bias, attrition bias, reporting bias and other bias. Each included study was rated as having a ‘low risk of bias’, ‘uncertain risk of bias’, or ‘high risk of bias’. As shown in Figure 2.

#### 3.1.3 Impact of FC on teaching indicator

**Figure 3:**
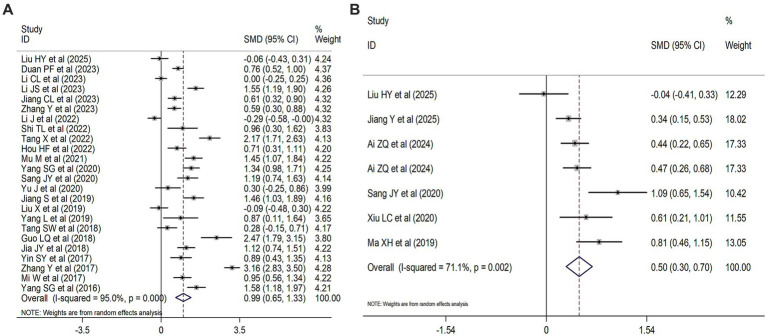
Forest plot of the meta-analysis for exam score. **(A)** Theoretical exam score; **(B)** Practical exam score.

Theoretical exam scores: A total of 24 studies (7, 10–20, 22–26, 28–34) compared students′ theoretical examination performance between the FC and LBL teaching modes. Cochran’s Q test indicated substantial heterogeneity across the included studies (*I^2^* = 95.0%, *p* < 0.001); therefore, a random-effects model was used for meta-analysis. The pooled SMD was 0.99 (95% CI: 0.65–1.33), and the difference is statistically significant (*p* < 0.05). As shown in Figure 3A.

Practical exam scores: For practical skills assessment, a total of 7 studies (7–9, 20, 21, 27) were included. Heterogeneity across these studies was moderate (*I^2^* = 71.1%, *p* = 0.002), and a random-effects model was used. The pooled SMD was 0.50 (95% CI: 0.30–0.70), and the difference was statistically significant (*p* < 0.05). As shown in Figure 3B.

**Figure 4:**
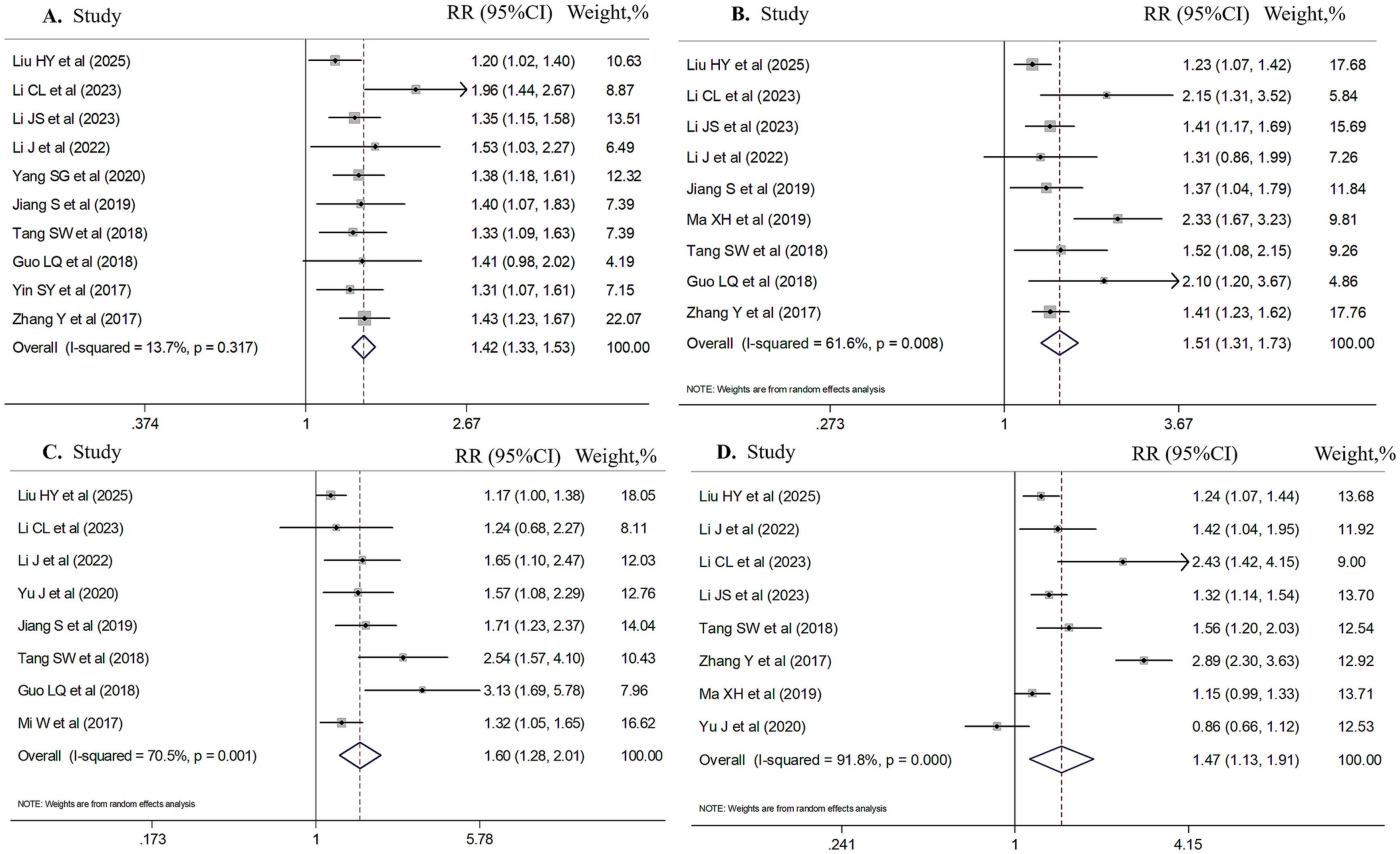
Forest plot of the meta-analysis for studied teaching index. **(A)** Learning interest. **(B)** Independent learning and thinking ability. **(C)** Cooperation and communication skills. **(D)** Ability to analyze and solve problems.

Learning interests: In the literature, a total of 10 studies (7, 11, 12, 15, 22, 25, 28, 29, 31, 33) compared students’ learning interests between FC and LBL teaching modes. Heterogeneity analysis showed low heterogeneity across the included study data (*I^2^* = 13.7%, *p* = 0.317). Therefore, a fixed-effect mode was used to pool the effect sizes. The results showed that the FC group had significantly greater learning interest than the LBL group, with a pooled RR of 1.42 (95% CI: 1.33–1.53), indicating that FC increased students’ learning interest by about 42%, as shown in Figure 4A.

Independent learning and thinking ability: Nine studies (7, 11, 12, 15, 25, 27–29, 31) compared students’ self-directed learning and independent thinking abilities between the FC and LBL teaching modes. Heterogeneity analysis showed significant heterogeneity across the included study data (*I^2^* = 61.6%, *p* = 0.008). Therefore, a random-effect model was used to pool the effect quantities. The meta-analysis showed that the FC group had significantly higher self-learning independent thinking ability than the LBL group, with a pooled RR of 1.51 (95% CI: 1.31–1.73), indicating that FC improved independent learning and thinking ability by about 52%, as shown in Figure 4B.

Collaborative Communication Skills: Eight studies (7, 11, 15, 23, 25, 28, 29, 32) compared students’ collaborative communication skills between FC and LBL teaching modes. Heterogeneity analysis showed significant heterogeneity across the included study data (*I^2^* = 70.5%, *p* = 0.001). Therefore, a random effects model is used to pool the effect sizes. The results showed that the cooperative communication ability of students in the FC group was significantly higher than that in the LBL group, with a pooled RR of 1.60 (95% CI: 1.28–2.01), see Figure 4C.

Ability to analyze and solve problems: Eight studies (7, 11, 12, 15, 23, 27, 28, 31) compared students’ analytical and problem-solving abilities between FC and LBL classroom teaching modes. Heterogeneity testing showed substantial heterogeneity across the studies (*I^2^* = 91.8%, *p* < 0.001). Therefore, a random effects model was used to pool the effect sizes. The results showed that students in the FC group had significantly higher analytical and problem-solving abilities than those in the LBL group, with a pooled RR of 1.47 (95% CI: 1.13–1.91), see Figure 4D.

Teacher-student interactions: Three studies (15, 25, 32) compared teacher-student interaction between FC and LBL teaching models. Heterogeneity testing showed no significant heterogeneity across the studies (*I^2^* < 0.1%, *p* = 0.952). Accordingly, a fixed-effect mode was used to pool the effect sizes. The results showed that teacher-student interaction in the FC group was significantly higher than that in the LBL group, with an RR of 1.35 (95% CI: 1.10–1.66). Due to the limited number of studies, the figure is not presented.

### 3.2 Analysis of publication bias

**Figure 5:**
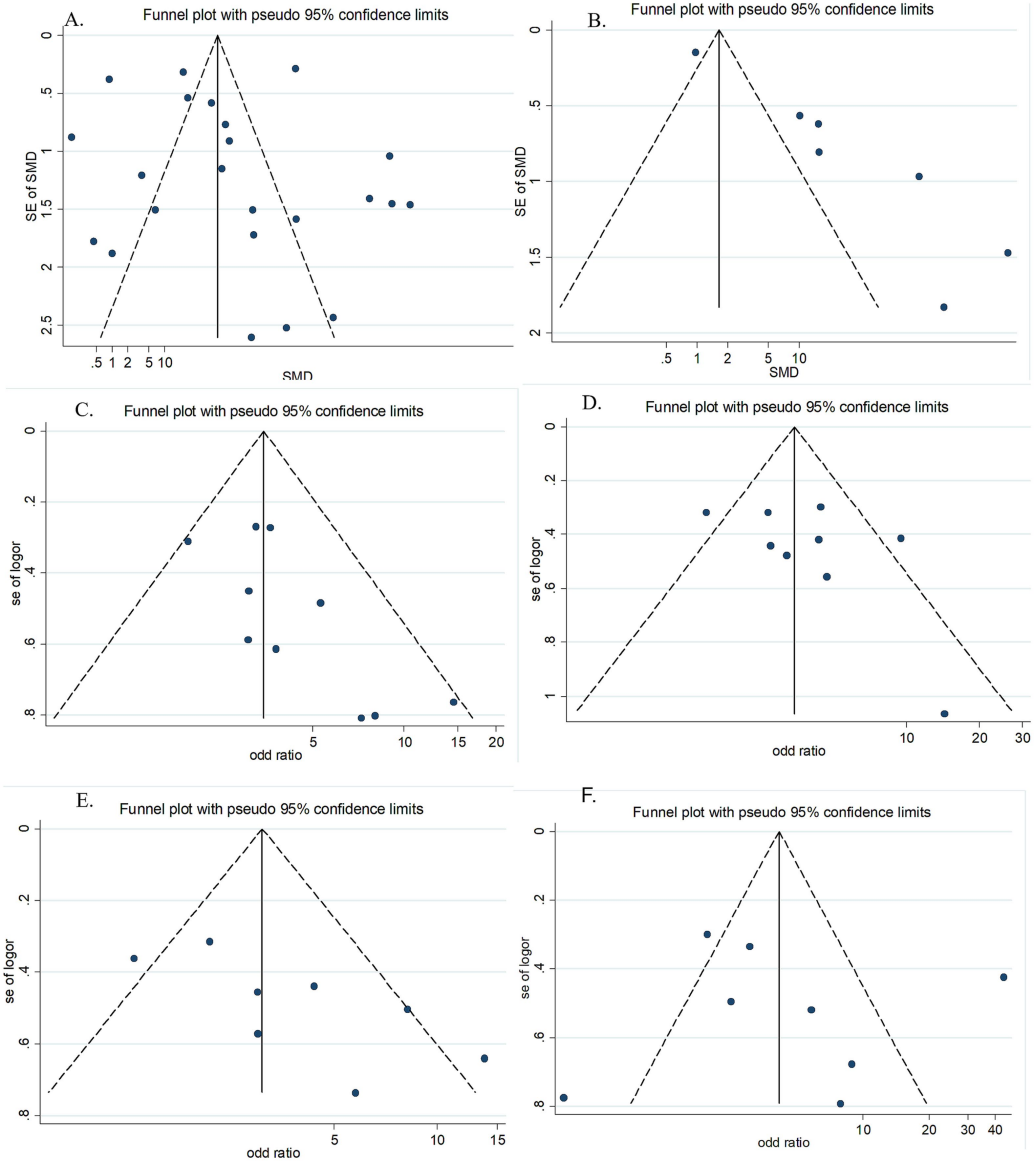
Funnel plot for publication bias assessment. **(A)** Theoretical exam score; **(B)** Practical exam score; **(C)** Learning interest; **(D)** Independent learning and thinking ability; **(E)** Cooperation and communication skills; **(F)** Ability to analyze and solve problems.

Begg’s test was used to assess publication bias. The results showed that the scatter of the included studies was roughly symmetrically distributed on both sides of the funnel plot’s symmetry axis. There was no significant publication bias for exam score (*P*_Begg’s_ = 0.760), cooperative communication ability (*P*_Begg’s_ = 0.063), independent learning and thinking ability (*P*_Begg’s_ = 0.466), analytical problem-solving ability (*P*_Begg’s_ = 0.536), and teacher-student interactions (*P*_Begg’s_ = 1.000, figure not shown). However, significant publication bias was detected for learning interest (*P*_Begg’s_ = 0.049). As shown in Figure 5. This suggests that the pooled result for learning interest may be influenced by small-study effects or a tendency to publish positive findings. Such bias may lead to an overestimation of the true effect size, and this finding should therefore be interpreted with caution.

### 3.3 Heterogeneity source analysis

To explore the detected heterogeneity across the included studies, meta-regression analysis was performed with publication year as a continuous covariate. The results showed that publication year may be one of a major source of heterogeneity for theoretical exam scores (t = −2.13, *p* = 0.045) and practical exam scores (t = −3.25, *p* = 0.023).

In addition, exploratory subgroup analyses stratified by trial group sample size (≤100 vs. >100) were conducted. The results indicated that sample size may be an important source of heterogeneity for practical exam scores and problem-solving ability. Compared with the overall analysis, heterogeneity was substantially reduced in the subgroup with an experimental group sample size > 100 (*I^2^* = 0%, *p* = 0.633 and *I^2^* = 0%, *p* = 0.558), respectively. See Supplementary Figure 1.

### 3.4 Sensitivity analysis

**Figure 6:**
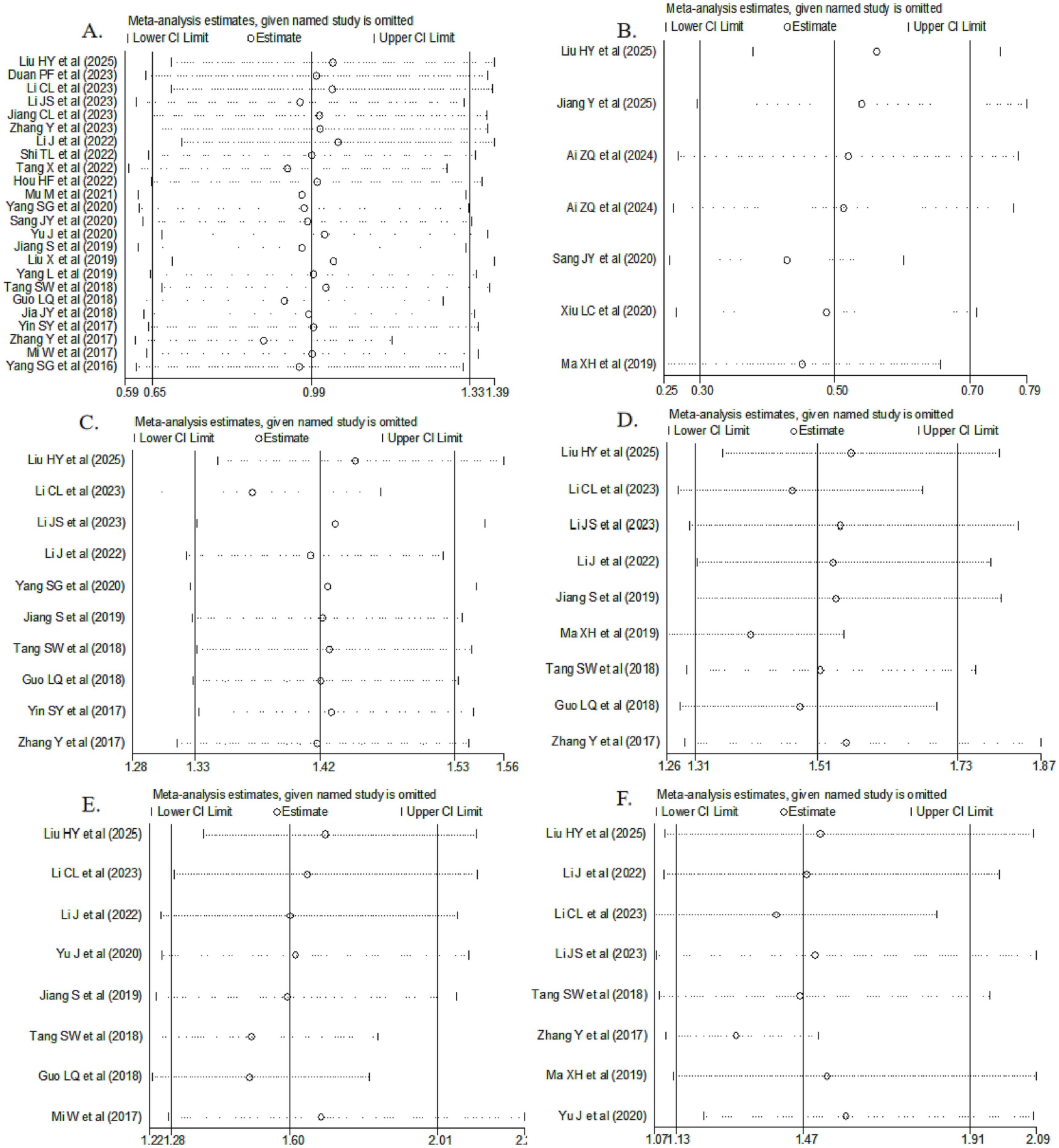
Leave-one-out sensitivity analysis. **(A)** Theoretical exam score; **(B)** Practical exam score; **(C)** Learning interest; **(D)** Independent learning and thinking ability; **(E)** Cooperation and communication skills; **(F)** Ability to analyze and solve problems.

Sensitivity analysis was performed using the leave-one-out method to assess the influence of individual studies on the pooled results. After iteratively removing each study, the 95% CI of the remaining studies remained completely to the right of 1 without crossing the null value, indicating no loss of statistical significance. The results were highly consistent with the overall analysis, and no single study was found to substantially influence the overall pooled estimates, demonstrating that the conclusions of this study have good reliability and robustness, as shown in Figure 6.

## 4 Discussion

This study systematically evaluated the educational effectiveness of FC in preventive medicine courses in China through meta-analysis. The results demonstrate that, compared with the LBL teaching model, FC not only significantly improves students’ theoretical exam scores (an objective indicators), but also effectively enhances students’ learning interest, self-learning and independent thinking ability, cooperative communication ability, analytical and problem-solving ability, and teacher-student interaction (subjective/ability-related indicators). This conclusion demonstrates good robustness, as validated by leave-one-out sensitivity analysis, suggesting that FC is an effective approach for curriculum reform in preventive medicine education.

We found that students in the FC group achieved significantly higher examination scores than those in the LBL group, which is consistent with the findings of the meta-analysis by Sui GY et al (35). Courses in preventive medicine, such as epidemiology and health statistics, are characterized by strong logical reasoning and abstract formulas, and traditional “spoon-feeding” instruction can easily result in students who “understand the concepts but cannot apply them” (1). By contrast, FC shifts knowledge delivery to before class (e.g., through previewing materials and micro-lecture videos), freeing up classroom time for case analysis, discussion, and discipline-related practice or exercises, which is in line with the “zone of proximal development” theory in cognitive psychology (36). This “learn first, teach later” model encourages students to engage in lower-order cognitive processes such as remembering and understanding before class, and to undertake higher-order cognitive processes such as analyzing, synthesizing, evaluating, and creating during subsequent classroom sessions, thereby achieving knowledge internalization and transfer, and ultimately leading to a significant improvement in assessment performance (37, 38).

In addition to academic performance, this study indicates that compared to traditional LBL teaching method, the FC model offers unique advantages in enhancing non-cognitive skills, which can be summarized as follows: (1) Stronger self-directed learning and independent thinking abilities. Preventive medicine emphasizes population perspective and evidence-based thinking, and FC gives students autonomy in controlling their learning progress and content, prompting them to shift from “passive receivers” to “active explorers,” thereby strengthening critical thinking (39, 40). Tomesko J et al. applied an FC model in an online graduate course in the United States and found that it facilitated active learning, provided immediate feedback, and enhanced participation, leading to more thorough information synthesis. They also concluded that it can improve students’ critical thinking skills (41, 42). (2) Improved collaborative communication and analytical problem-solving skills. This study found that FC teaching significantly enhanced these two indicators. In the group task of FC, students need to simulate an on-site public health investigation and engage in team collaboration. This problem-based learning environment simulates real-world public health work scenarios. This can be explained by the fact that teamwork is a core competency for solving complex public health problems, and FC provides precisely this training ground. (3) Enhanced teacher-student interaction. In traditional large class teaching, it is difficult for teachers to address individual differences, whereas FC transforms the role of teachers from “sage on the podium” to “guide at their side,” enabling precise guidance on common problems identified during students’ pre-class preparation and thereby facilitating mutual learning and teaching (43, 44).

It is worth noting that this study found that publication bias was mainly concentrated in the indicators of “learning interest.” This may be attributable to two factors: firstly, the measurement of subjective indicators relies heavily on self-reported questionnaires, which is susceptible to the Hawthorne Effect and social desirability bias, and there is a greater tendency for studies with positive results to be published (45); Secondly, small-sample studies that do not show significant differences may remain unpublished due to editorial preference. However, sensitivity analysis showed that after excluding any individual study, the combined effect size did not undergo a substantial reversal. This suggests that, despite the presence of some publication bias, the overall conclusions of this study are robust, are not unduly influenced by extreme individual studies, and have high credibility.

Although this study confirms the effectiveness of FC teaching in preventive medicine, there are still limitations. First, all included studies were conducted in China, which restricts the generalizability of our findings to other educational contexts and populations. Second, the lack of standardized outcome measures across studies, particularly for subjective indicators such as learning interest and interaction, may have introduced heterogeneity and potential measurement bias. Third, the generally small sample sizes of included studies raise concerns about small-study effects, which may overestimate the true effect size. Moreover, although a random-effects model was adopted, it is difficult to fully account for the variability arising from differences in teaching implementation. Finally, the review protocol was not prospectively registered in PROSPERO, which may introduce potential reporting bias and limit the reproducibility of our methodological decisions, despite adherence to PRISMA reporting standards.

In summary, within the context of undergraduate preventive medicine education in China, the FC approach appears to offer beneficial effects, which may contribute to improving students’ academic performance and potentially fostering their core competencies as public health practitioners. However, these findings should be interpreted with caution. It is suggested that medical colleges could consider promoting this model according to their specific circumstances, while also emphasizing the development of teachers’ information technology teaching abilities to ensure the effectiveness of the reform.

## Data Availability

The original contributions presented in the study are included in the article/Supplementary material, further inquiries can be directed to the corresponding authors.
